# (*Z*)-2-Hydr­oxy-3-(4-methoxy­phen­yl)acrylic acid

**DOI:** 10.1107/S1600536809050077

**Published:** 2009-11-28

**Authors:** Hui Ouyang, Qi-Jian Tian, Yong-Dong Jiang, Chun-Lian Tian

**Affiliations:** a Key Laboratory of Hunan Forest Products and Chemical Industry Engineering, Jishou University, Jishou 416000, People’s Republic of China

## Abstract

In the structure of the title compound, C_10_H_10_O_4_, inversion dimers linked by pairs of O—H⋯O hydrogen bonds link the carboxylic acid groups. Further O—H⋯O links cross-link the dimers into sheets running along the *b*-axis direction.

## Related literature

For 3-phenyl­acrylic acid as inter­mediates for compounds with biological activity, see: Chen *et al.* (1993 ▶); Igarashi *et al.* (1997 ▶); Xiao *et al.* (2007 ▶); Yu *et al.* (1991 ▶). The title compound was synthesized during the course of our work on the synthesis of potential anti­cancer compounds, see: Xiao *et al.* (2008*a* ▶,*b* ▶).
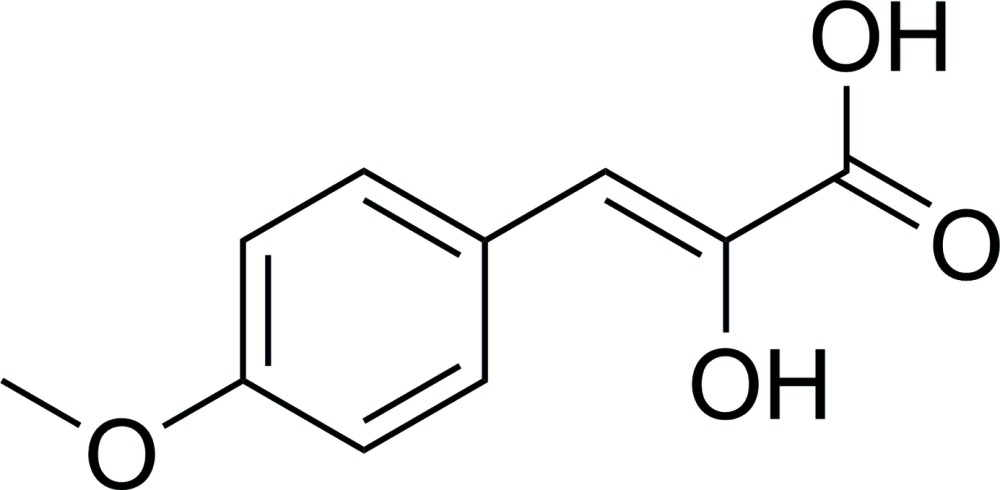


## Experimental

### 

#### Crystal data

C_10_H_10_O_4_
*M* *_r_* = 194.18
Monoclinic, 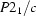

*a* = 6.7440 (13) Å
*b* = 5.4290 (11) Å
*c* = 24.933 (5) Å
β = 93.28 (3)°
*V* = 911.4 (3) Å^3^
*Z* = 4
Mo *K*α radiation
μ = 0.11 mm^−1^
*T* = 298 K
0.20 × 0.10 × 0.05 mm

#### Data collection

Bruker SMART APEX CCD diffractometer
Absorption correction: multi-scan (*SADABS*; Sheldrick, 1996 ▶) *T*_min_ = 0.978, *T*_max_ = 0.995
1783 measured reflections
1634 independent reflections
1062 reflections with *I* > 2σ(*I*)
*R*_int_ = 0.025

#### Refinement

*R*[*F* ^2^ > 2σ(*F* ^2^)] = 0.079
*wR*(*F* ^2^) = 0.229
*S* = 1.07
1634 reflections
127 parameters
H-atom parameters constrained
Δρ_max_ = 0.34 e Å^−3^
Δρ_min_ = −0.25 e Å^−3^

### 

Data collection: *SMART* (Bruker, 2007 ▶); cell refinement: *SAINT* (Bruker, 2007 ▶); data reduction: *SAINT*; program(s) used to solve structure: *SHELXS97* (Sheldrick, 2008 ▶); program(s) used to refine structure: *SHELXL97* (Sheldrick, 2008 ▶); molecular graphics: *SHELXTL* (Sheldrick, 2008 ▶); software used to prepare material for publication: *SHELXL97*.

## Supplementary Material

Crystal structure: contains datablocks global, I. DOI: 10.1107/S1600536809050077/jh2116sup1.cif

Structure factors: contains datablocks I. DOI: 10.1107/S1600536809050077/jh2116Isup2.hkl

Additional supplementary materials:  crystallographic information; 3D view; checkCIF report

## Figures and Tables

**Table 1:** Hydrogen-bond geometry (Å, °)

*D*—H⋯*A*	*D*—H	H⋯*A*	*D*⋯*A*	*D*—H⋯*A*
O3—H3*B*⋯O4^ii^	0.85	1.78	2.626 (4)	177

Symmetry codes: (i) 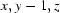; (ii) 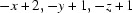.

**Fig. 1.**
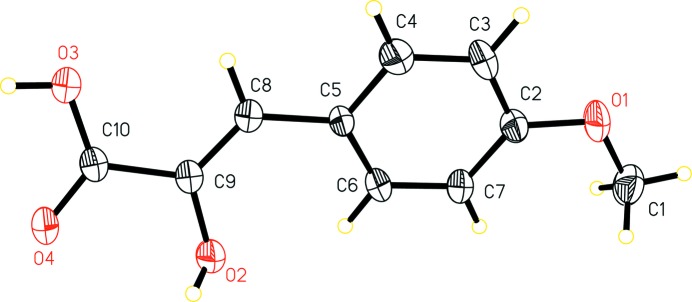
The independent components of (I), showing the atom-labelling scheme. Displacement ellipsoids are drawn at the 30% probability level. Dashed lines indicate hydrogen bonds.

**Fig. 2.**
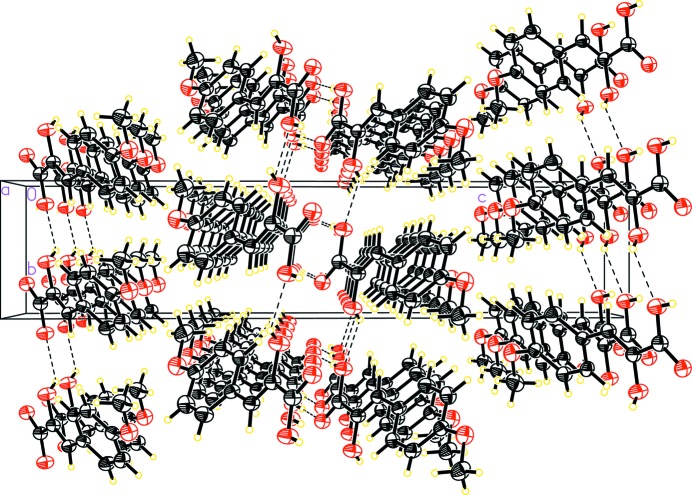
Pattern forms through intermolecular O—H···O hydrogen bonds. Dashed lines indicate hydrogen bonds.

## supplementary crystallographic information

### Comment

Derivatives of 3-phenylacrylic acid are key intermediates for tanshinol (Yu *et al.* 1991), resormycin (Igarashi *et al.* 1997; Xiao *et al.* 2007) and benzylazauracil (Chen *et al.* 1993), which show anti-platelet aggregation, antifungal and antiviral activities, respectively. In the course of our work on screening for anticancers (Xiao, *et al.* 2008*a*; Xiao, *et al.* 2008*b*), we synthesized the title compound and herein reported its crystal structure.

In the title compound (I), (*Z*)-2-hydroxy-3-(4-methoxyphenyl)acrylic acid, the plane of benzene ring (with mean dieviation deviation of 0.0053 Å) and the plane of hydroxy acrylic moiety (with mean deviation of 0.0049 Å) make a dihedral angle of 18.001 (97) Å. The benzene ring and the carboxy group occur on opposite side of the C8═C9 double bond with torsion angle of 179.8 (4) ° (Fig. 1). The molecules are linked into dimers by the intermolecular hydrogen bonds occurring the carboxylic acid groups, which lie on crystallographic centres of inversion. These dimers are further cross-linked by intermolecular hydrogen bonds between enolic hydroxy groups and carboxylic acid groups to form sheets running parallel to the crystallographic *b* axis direction (Table 1 and Fig. 2).

### Experimental

The mixture of alpha-acetoamido-4-methoxycinnamic acid (2.35 g, 10 mmol) in 0.5*M* HCl (60 mL) was refluxed for 6 h. The resulting mixture was allowed to cool to room temperature and the resulting precipitate was collected by filtration. The crude product was dissolved in EtOAc and twofold volume of petroleum was added carefully. Colorless blocks of (I) suitable for single-crystal structure determination was furnished after 2 d.

### Refinement

All H atoms were placed in geometrically idealized positions and constrained to ride on their parent atoms with C—H of 0.93 Å for the aromatic H atoms and CH groups, 0.96 Å for the CH_3_ groups and with O—H of 0.82 Å for the OH groups. *U*_iso_(H) values were set at 1.2 times *U*_eq_(C) for aromatic C groups, 1.5 times *U*_eq_(C) for CH_3_, 1.2 times *U*_eq_(O) for enolic O—H groups and 1.5 times *U*_eq_(O) for carboxylic O—H groups.

### Crystal data

**Table:**

C_10_H_10_O_4_	*F*(000) = 408
*M**_r_* = 194.18	*D*_x_ = 1.415 Mg m^−^^3^
Monoclinic, *P*2_1_/*c*	Mo *K*α radiation, λ = 0.71073 Å
Hall symbol: -P 2ybc	Cell parameters from 25 reflections
*a* = 6.7440 (13) Å	θ = 10–13°
*b* = 5.4290 (11) Å	µ = 0.11 mm^−^^1^
*c* = 24.933 (5) Å	*T* = 298 K
β = 93.28 (3)°	Block, colorless
*V* = 911.4 (3) Å^3^	0.20 × 0.10 × 0.05 mm
*Z* = 4	

### Data collection

**Table:**

Bruker SMART APEX CCD diffractometer	1634 independent reflections
Radiation source: fine-focus sealed tube	1062 reflections with *I* > 2σ(*I*)
graphite	*R*_int_ = 0.025
φ and ω scan	θ_max_ = 25.2°, θ_min_ = 1.6°
Absorption correction: multi-scan (*SADABS*; Sheldrick, 1996)	*h* = −8→8
*T*_min_ = 0.978, *T*_max_ = 0.995	*k* = 0→6
1783 measured reflections	*l* = 0→29

### Refinement

**Table:**

Refinement on *F*^2^	Primary atom site location: structure-invariant direct methods
Least-squares matrix: full	Secondary atom site location: difference Fourier map
*R*[*F*^2^ > 2σ(*F*^2^)] = 0.079	Hydrogen site location: inferred from neighbouring sites
*wR*(*F*^2^) = 0.229	H-atom parameters constrained
*S* = 1.07	*w* = 1/[σ^2^(*F*_o_^2^) + (0.1176*P*)^2^ + 0.6125*P*] where *P* = (*F*_o_^2^ + 2*F*_c_^2^)/3
1634 reflections	(Δ/σ)_max_ < 0.001
127 parameters	Δρ_max_ = 0.34 e Å^−^^3^
0 restraints	Δρ_min_ = −0.25 e Å^−^^3^

### Special details

**Table:**

Geometry. All e.s.d.'s (except the e.s.d. in the dihedral angle between two l.s. planes) are estimated using the full covariance matrix. The cell e.s.d.'s are taken into account individually in the estimation of e.s.d.'s in distances, angles and torsion angles; correlations between e.s.d.'s in cell parameters are only used when they are defined by crystal symmetry. An approximate (isotropic) treatment of cell e.s.d.'s is used for estimating e.s.d.'s involving l.s. planes.
Refinement. Refinement of *F*^2^ against ALL reflections. The weighted *R*-factor *wR* and goodness of fit *S* are based on *F*^2^, conventional *R*-factors *R* are based on *F*, with *F* set to zero for negative *F*^2^. The threshold expression of *F*^2^ > σ(*F*^2^) is used only for calculating *R*-factors(gt) *etc*. and is not relevant to the choice of reflections for refinement. *R*-factors based on *F*^2^ are statistically about twice as large as those based on *F*, and *R*- factors based on ALL data will be even larger.

### Fractional atomic coordinates and isotropic or equivalent isotropic displacement parameters (Å^2^)

**Table:**

	*x*	*y*	*z*	*U*_iso_*/*U*_eq_	
O1	−0.2792 (4)	0.2913 (6)	0.29127 (12)	0.0686 (10)	
C1	−0.3926 (7)	0.0765 (10)	0.2992 (2)	0.0735 (14)	
H1A	−0.5117	0.0816	0.2762	0.110*	
H1B	−0.4268	0.0681	0.3360	0.110*	
H1C	−0.3163	−0.0662	0.2907	0.110*	
O2	0.5210 (4)	0.0927 (5)	0.44683 (13)	0.0627 (9)	
H2A	0.6147	0.0066	0.4352	0.075*	
C2	−0.1051 (6)	0.3189 (8)	0.32160 (16)	0.0511 (10)	
O3	0.7993 (4)	0.6459 (5)	0.45988 (12)	0.0625 (9)	
H3B	0.9134	0.6691	0.4754	0.094*	
C3	0.0063 (6)	0.5256 (9)	0.30978 (18)	0.0641 (13)	
H3A	−0.0385	0.6312	0.2823	0.077*	
O4	0.8501 (4)	0.2652 (5)	0.49281 (11)	0.0554 (8)	
C4	0.1819 (7)	0.5746 (9)	0.33834 (18)	0.0622 (12)	
H4A	0.2529	0.7155	0.3304	0.075*	
C5	0.2557 (5)	0.4185 (7)	0.37875 (15)	0.0461 (9)	
C6	0.1417 (6)	0.2101 (8)	0.38927 (16)	0.0517 (10)	
H6A	0.1882	0.1006	0.4158	0.062*	
C7	−0.0345 (6)	0.1624 (8)	0.36199 (17)	0.0552 (11)	
H7A	−0.1077	0.0241	0.3705	0.066*	
C8	0.4431 (6)	0.4777 (7)	0.40817 (15)	0.0484 (10)	
H8A	0.4839	0.6407	0.4056	0.058*	
C9	0.5639 (5)	0.3336 (7)	0.43806 (16)	0.0479 (9)	
C10	0.7504 (5)	0.4146 (8)	0.46618 (16)	0.0483 (10)	

### Atomic displacement parameters (Å^2^)

**Table:**

	*U*^11^	*U*^22^	*U*^33^	*U*^12^	*U*^13^	*U*^23^
O1	0.0498 (16)	0.078 (2)	0.074 (2)	−0.0062 (16)	−0.0308 (15)	0.0012 (17)
C1	0.057 (2)	0.077 (3)	0.084 (3)	−0.006 (3)	−0.019 (2)	−0.015 (3)
O2	0.0473 (15)	0.0473 (17)	0.090 (2)	−0.0052 (13)	−0.0239 (15)	0.0071 (16)
C2	0.0456 (19)	0.051 (2)	0.055 (2)	0.0079 (19)	−0.0146 (18)	−0.0052 (19)
O3	0.0499 (16)	0.0494 (17)	0.085 (2)	−0.0063 (14)	−0.0251 (15)	0.0020 (15)
C3	0.053 (2)	0.070 (3)	0.065 (3)	0.008 (2)	−0.027 (2)	0.008 (2)
O4	0.0408 (14)	0.0575 (18)	0.0655 (17)	−0.0033 (14)	−0.0187 (13)	0.0021 (15)
C4	0.056 (2)	0.054 (3)	0.075 (3)	−0.002 (2)	−0.013 (2)	0.008 (2)
C5	0.0430 (19)	0.0406 (19)	0.052 (2)	0.0067 (18)	−0.0200 (17)	−0.0055 (18)
C6	0.049 (2)	0.052 (2)	0.051 (2)	0.0037 (19)	−0.0200 (17)	0.0046 (19)
C7	0.045 (2)	0.052 (2)	0.066 (3)	−0.004 (2)	−0.0200 (19)	−0.002 (2)
C8	0.044 (2)	0.044 (2)	0.055 (2)	−0.0037 (18)	−0.0130 (18)	−0.0002 (18)
C9	0.0402 (19)	0.046 (2)	0.056 (2)	−0.0004 (18)	−0.0125 (17)	−0.0007 (18)
C10	0.0343 (17)	0.049 (2)	0.060 (2)	0.0007 (18)	−0.0080 (17)	0.000 (2)

### Geometric parameters (Å, °)

**Table:**

O1—C2	1.368 (5)	C3—H3A	0.9300
O1—C1	1.415 (6)	O4—C10	1.225 (4)
C1—H1A	0.9600	C4—C5	1.387 (6)
C1—H1B	0.9600	C4—H4A	0.9300
C1—H1C	0.9600	C5—C6	1.401 (6)
O2—C9	1.360 (5)	C5—C8	1.460 (5)
O2—H2A	0.8500	C6—C7	1.360 (5)
C2—C7	1.381 (6)	C6—H6A	0.9300
C2—C3	1.391 (6)	C7—H7A	0.9300
O3—C10	1.310 (5)	C8—C9	1.327 (5)
O3—H3B	0.8499	C8—H8A	0.9300
C3—C4	1.372 (6)	C9—C10	1.472 (5)
			
C2—O1—C1	117.8 (3)	C4—C5—C6	116.9 (3)
O1—C1—H1A	109.5	C4—C5—C8	119.7 (4)
O1—C1—H1B	109.5	C6—C5—C8	123.5 (3)
H1A—C1—H1B	109.5	C7—C6—C5	122.2 (4)
O1—C1—H1C	109.5	C7—C6—H6A	118.9
H1A—C1—H1C	109.5	C5—C6—H6A	118.9
H1B—C1—H1C	109.5	C6—C7—C2	120.2 (4)
C9—O2—H2A	107.7	C6—C7—H7A	119.9
O1—C2—C7	125.8 (4)	C2—C7—H7A	119.9
O1—C2—C3	115.4 (4)	C9—C8—C5	129.7 (4)
C7—C2—C3	118.8 (4)	C9—C8—H8A	115.2
C10—O3—H3B	108.4	C5—C8—H8A	115.2
C4—C3—C2	120.5 (4)	C8—C9—O2	121.9 (3)
C4—C3—H3A	119.8	C8—C9—C10	124.9 (4)
C2—C3—H3A	119.8	O2—C9—C10	113.2 (3)
C3—C4—C5	121.4 (4)	O4—C10—O3	124.4 (3)
C3—C4—H4A	119.3	O4—C10—C9	119.2 (4)
C5—C4—H4A	119.3	O3—C10—C9	116.3 (3)
			
C1—O1—C2—C7	−4.2 (6)	O1—C2—C7—C6	179.9 (4)
C1—O1—C2—C3	176.1 (4)	C3—C2—C7—C6	−0.4 (6)
O1—C2—C3—C4	178.8 (4)	C4—C5—C8—C9	−161.8 (4)
C7—C2—C3—C4	−0.9 (7)	C6—C5—C8—C9	18.8 (7)
C2—C3—C4—C5	1.4 (7)	C5—C8—C9—O2	−1.9 (7)
C3—C4—C5—C6	−0.5 (7)	C5—C8—C9—C10	−179.8 (4)
C3—C4—C5—C8	−180.0 (4)	C8—C9—C10—O4	179.8 (4)
C4—C5—C6—C7	−0.9 (6)	O2—C9—C10—O4	1.8 (5)
C8—C5—C6—C7	178.5 (4)	C8—C9—C10—O3	−1.2 (6)
C5—C6—C7—C2	1.4 (7)	O2—C9—C10—O3	−179.3 (4)

### Hydrogen-bond geometry (Å, °)

**Table:**

*D*—H···*A*	*D*—H	H···*A*	*D*···*A*	*D*—H···*A*
C6—H6A···O2	0.93	2.33	2.931 (5)	122
C8—H8A···O3	0.93	2.46	2.813 (5)	103
O2—H2A···O3^i^	0.85	2.38	3.073 (4)	139
O3—H3B···O4^ii^	0.85	1.78	2.626 (4)	177

Symmetry codes: (i) *x*, *y*−1, *z*; (ii) −*x*+2, −*y*+1, −*z*+1.
